# Eukaryotic initiation factor 4E: a key factor of traumatic stress-induced depression-related cognitive decline at different age

**DOI:** 10.1038/s41398-026-03860-7

**Published:** 2026-02-12

**Authors:** Chi-Wei Lee, Tzu-Jung Yang, Ming-Chia Chu, Kuen-Haur Lee, Tin Hoang Nguyen, Cheng-Ta Li, Hui-Ching Lin

**Affiliations:** 1 Brain Research Center, National Yang Ming Chiao Tung University, Taipei, Taiwan; 2 Department and Institute of Physiology, College of Medicine, National Yang Ming Chiao Tung University, Taipei, Taiwan; 3 Graduate Institute of Cancer Biology and Drug Discovery, College of Medical Science and Technology, Taipei Medical University, Taipei, Taiwan; 4 Department of Physiology, Faculty of Medicine, Can Tho University of Medicine and Pharmacy, Can Tho, Vietnam; 5 Department of Functional Exploration, Can Tho University of Medicine and Pharmacy Hospital, Can Tho, Vietnam; 6 Department of Psychiatry, Taipei Veterans General Hospital, Taipei, Taiwan; 7 Institute of Brain Science, National Yang Ming Chiao Tung University, Taipei, Taiwan; 8 Division of Psychiatry, Faculty of Medicine, National Yang Ming Chiao Tung University, Taipei, Taiwan; 9 Membrane Protein Structural Biology Research Center, National Yang Ming Chiao Tung University, Taipei, Taiwan; 10 Ph.D. Program in Medical Neuroscience, College of Medical Science and Technology, Taipei Medical University, Taipei, Taiwan

**Keywords:** Molecular neuroscience, Long-term memory

## Abstract

Depression in patients with cognitive impairment may be a risk factor for dementia. A previous study demonstrated that patients with treatment-resistant depression (TRD), characterized by poor response to adequate antidepressant treatment, exhibit pronounced cognitive impairment. Although depression in midlife or later life are associated with an increased risk of developing dementia, the mechanisms linking cognitive impairment and midlife depression remain poorly understood. A recent study revealed that downregulation of the γ-aminobutyric acid mediated (GABAergic) system and increased phosphorylation of eukaryotic translation initiation factor (eIF4E) are strongly associated with cognitive dysfunction and depressive symptoms. Hence, we hypothesized that the GABAergic system and eIF4E phosphorylation are involved in cognitive dysfunction and depression, particularly forms resistant to treatment, with increased aging. In the present study, we used a mouse model exhibiting antidepressant-resistant symptoms induced by traumatic stress in young- and middle-aged mice. Depression-like behavior was observed after traumatic stress exposure in both young and middle-aged mice. However, cognitive impairment induced by traumatic stress was worse in middle-aged mice. Moreover, the expression of GABA_A_ and GABA_B_ receptors decreased after traumatic stress exposure in both young and middle-aged mice. However, the increase in eIF4E phosphorylation was greater after traumatic stress exposure in the middle-aged mice. Depressive-like behavior was improved by inhibition of eIF4E phosphorylation and GABA receptor activation, whereas cognitive impairment was only improved by inhibition of eIF4E phosphorylation. These findings suggest that eIF4E plays a key role in age-related cognitive dysfunction and depression.

## Introduction

Treatment-resistant depression (TRD), characterized by a lack of adequate response to treatment with two or more antidepressants, represents a considerable challenge in psychiatry. Moreover, cognitive impairment is strongly associated with depressive symptoms [1]. Individuals with late-life depression had a 3.9 times higher risk of developing dementia than those without a history of depression [2]. In particular, cognitive impairment is a significant problem in patients with TRD [3]. A clinical study found that late-life depression, particularly TRD, significantly increases cognitive dysfunction and Aβ deposits [4]. Furthermore, the greater depressive symptom severity is associated with poorer cognitive function in patients with midlife depression [5]. A study on trajectories of depressive symptoms also found that persistently high depressive symptom trajectories may indicate an increased risk of developing dementia [6]. These studies highlight the need to investigate the relationship between midlife depression and cognitive function to develop preventive strategies aimed at reducing the risk of developing dementia. Cognitive dysfunction is often associated with depression and a poor response to antidepressant treatment [7]. However, there is limited understanding of the key mechanisms linking midlife depression to cognitive impairment and subsequent development of dementia.

The dysfunction of glutamatergic and γ-aminobutyric acid mediated (GABAergic) neurotransmission contributes in depression and age-related cognitive decline [8, 9]. A postmortem study indicated that GABA_A_ receptor (GABA_A_R) subunit levels are associated with depression [10]. Decreased α1, α3, β2, and γ1 subunit levels of the GABA_A_R within the prefrontal cortex (PFC) were found in patients with depression [11, 12]_._ An electroencephalography study found lower GABA_A_R-mediated activity and GABA_B_ receptor (GABA_B_R)-mediated activity in patients with depression [13]. Furthermore, magnetic resonance spectroscopy revealed an age-related decline in the concentrations of both GABA and glutamate in the PFC, beginning in midlife [14]. Lower expression of the GABA_A_R α5 and GABA_B_R B1 subunits in the hippocampus of aged-rats has also been reported [15], and is associated with spatial memory impairment [16]. Significantly reduced GABA_B_R-mediated inhibitory tone has been reported in the PFC of aged animals [17], suggesting that the GABAergic system is highly associated with depression and age-related cognitive impairment.

The antidepressant effect of low-dose ketamine, an N-methyl-D-aspartic acid receptor antagonist, involves enhanced eukaryotic elongation factor 2 phosphorylation and brain-derived neurotrophic factor (BDNF) synthesis [18], as well as mammalian target of rapamycin (mTOR)-mediated phosphorylation of eukaryotic translation initiation factor 4E binding protein (4E-BP) [19, 20]. Notably, eukaryotic translation initiation factor (eIF4E) phosphorylation, which regulates translation via 4E-BPs, is also critical for cognitive function, as mice lacking this phosphorylation exhibit cognitive impairment [21]. Interestingly, eIF4E phosphorylation was considerably elevated in postmortem brains of patients with Alzheimer’s disease (AD) [22]. Additionally, eIF4E is involved in the antidepressant effect of selective serotonin reuptake inhibitors, and loss of eIF4E phosphorylation in mice results in reduced levels of serotonin, depression, and anxiety-like behaviors [23]. Therefore, eIF4E phosphorylation may play a critical role in depression and age-related cognitive impairment.

Given that stress is a major precipitating factor in the pathophysiology of depression and is frequently associated with a history of trauma and comorbid post-traumatic stress disorder (PTSD) [24], we sought to establish an animal model that captures the core features of TRD. Epidemiological studies have demonstrated a significantly increased risk of depressive disorder in individuals with PTSD [25]. According to the Sequenced Treatment Alternatives to Relieve Depression (STAR*D) trial, approximately 22.4% of patients with TRD exhibited comorbid PTSD [26], highlighting the close clinical association between chronic trauma and resistance to antidepressant treatment.

In order to evaluate the impact of early age onset depression on cognitive function, we applied tone and foot-shock pairings as traumatic stress to induce a poor antidepressant response phenotype in young and middle-aged mice to determine whether the eIF4E or GABAergic system is involved in the association between depression and age-related cognitive function.

## Materials and methods

### Animals

Male C57BL/6 J adult mice, aged 8 weeks (young adult) and 40 weeks (late adult) were purchased from the National Laboratory Animal Center (Taipei, Taiwan). Mice were group-housed under a 12-h dark/light cycle at constant temperature (24 °C), with free access to water and food. All animals were maintained in accordance with the recommendations of the Guide for the Care and Use of Laboratory Animals (National Institute for Laboratory Animal Research, USA). All the procedures were approved by the Institutional Animal Care and Use Committee (IACUC) of National Yang Ming Chiao Tung University Animal Core Facility in Taipei, Taiwan (IACUC No. 1120106rr and 1130309rr).

#### Fear conditioning

The mice were placed in a standard operant chamber (Coulbourn Instruments, Whitehall, PA, USA) equipped with stainless-steel grid floors connected to a shock generator for foot-shock delivery. The auditory conditioned stimulus (CS) consisted of white noise at 95 dB, while the unconditioned stimulus (US) was a 0.8-mA foot-shock. During the two-day training period, following a 120-s habitation period, the mice received ten CS–US pairings. Each pairing involved a 20-s CS, co-terminating with a 3-s US, with a 60-s inter-trial interval (ITI). This procedure was repeated over two consecutive days to induce traumatic stress. After 24 h of training, the mice were returned to the same chamber for a fear memory test. Following a 120-s habituation period, the mice were exposed to either three presentations of the auditory tone alone (CS) or no tone (Non-CS). Freezing behavior was defined as the absence of any movement except respiration and was measured automatically using Graphic state software (Coulbourn Instruments).

#### Sucrose preference test

The sucrose preference test (SPT) was employed to assess anhedonia behavior in mice following 1 week of traumatic stress exposure. Prior to testing, mice were housed individually in home cages with ad libitum access to food and two bottles of regular tap water for overnight habituation. On the test day, mice underwent water restriction for 2-h prior to test. During the test, mice were provided with free access to a bottle of 2.5% sucrose solution and another containing regular tap water for 4 h. Sucrose preference was calculated as the percentage of sucrose solution intake relative to the total volume consumption.

#### Tail suspension test

The tail suspension test (TST) was conducted to evaluate depressive-like behavior in mice following 1 week of traumatic stress exposure. Mice were individually suspended the tail using adhesive tape affixed approximately 1 cm from the tip, and attached to a horizontal ring-stand bar (20 cm in diameter, 40 cm in height). Each session lasted 5 min, and the immobility time was defined as the mice hung passively and completely motionless.

#### Forced swim test

The forced swim test (FST) was employed to access the active coping behavior mice following one week of traumatic stress exposure. Mice were individually placed in a transparent cylindrical container (15 cm in diameter and 30 cm in height) filled with 20 cm of water (25 ± 1 °C). Each session lasted 5 min, and the water was replaced between trials. The immobility time was defined as the complete absence of movement in the mice.

#### Female urine sniffing test

The female urine sniffing test (FUST) was conducted to evaluate reward-seeking activity in mice following one week of traumatic stress exposure. In the initial phase, each mouse was placed in a cage containing a cotton swab moistened in regular tap water for 3 min of free exploration. Approximately 45 min later, the same mouse was placed in a cage with a cotton swab moistened with fresh female urine (collected from age- and strain-matched female mice) for 3 min of free exploration. The duration and frequency of sniffing directed toward the cotton swab were recorded.

#### Spontaneous alteration Y-maze

The Spontaneous alteration Y-maze was routinely used to evaluate the spatial working memory of testing in mice following 1 week of traumatic stress exposure. The Y-maze consisted of three symmetrical and identical arms (32 cm in length and 15 cm in wide with 9 cm height). Mice were placed within the end of one arm for 8 min of free exploration, and the sequence and number of arm entries were recorded. A spontaneous alternation was defined as the mice exploring all three arms (i.e., ABC, CAB, or BCA but not repeated arms such as ABB). The alternation score was calculated as the ratio of the actual number of alternations to the possible number (defined as the total number of arm entries minus two), according to the following formula:

$$\mathrm{Alternation}\, \% =[(\mathrm{Number}\,\mathrm{of}\,\mathrm{alternations})/(\mathrm{Total}\,\mathrm{arm}\,\mathrm{entries}-2)]* 100.$$

#### Novel object recognition task

The Novel Object Recognition Task (NORT) was used to assess the recognition ability of the mice following 1 week of traumatic stress exposure. A cuboid plastic chamber (45*45*45 cm) was first prepared. On habituation day, the mice were placed in the center of the chamber and allowed to explore freely for 10 min. The chamber was cleaned with 75% ethanol between each trial. On training day, two identical objects (acrylic bottles each measuring 5.5 cm in diameter and 14 cm in height) were placed in the diagonal corner of the chamber. Mice were placed in the center of the chamber and explore freely for 10 min. The accumulated time was recorded when the mouse approached the objects within a 2 cm distance and explored the two objects for a total of 60 seconds. On testing day, the object was replaced by a new object (plastic ball 6 cm in diameter). Mice were placed in the center of the chamber again and the time of exploration of the old (familiar) vs. new (novel) object was recorded until the total exploration time of these two objects reached 60 s. The discrimination index (DI) was determined, based on the following formula: DI = (time of novel−familiar)/ (time of novel +familiar).

### Depressive-emotional score and cognitive-discrimination score calculation

The depressive-emotional score and cognitive-discrimination score were calculated using the Z-score dimensionless mathematical tools to capture behavioral variability and achieve comprehensive, integrated metrics within each group [27]. The Z-score of individual animals for each behavior were calculation using the formula below, which indicated how many standard deviations (σ) an observation (X) is above or below the mean of a control group (μ).

$${\rm{Z}}=\frac{X-\mu }{\sigma }$$

The individual depressive-emotional score and cognitive-discrimination score were calculated by averaging Z-scores within the tests, and then across the tests to ensure equal weighting of the depressive-related behaviors or cognitive-related behaviors comprising the final Z-score.

### Brain slice preparation and electrophysiology recording

The mice were sacrificed by rapid decapitation, and the brains were quickly removed and immersed in a beaker containing cold (4 °C) oxygenated artificial cerebrospinal fluid (aCSF) saturated with 95% O_2_ and 5% CO_2_. The aCSF contained 117 mM NaCl, 4.7 mM KCl, 1.2 mM MgCl_2_, 1.2 mM NaHCO_3_, 2.5 mM CaCl_2_, 25 mM NaHCO_3_, and 11 mM glucose. Coronal brain slices (400 μm thick) were prepared using a vibratome. To record the field excitatory post-synaptic potential (fEPSP) in the PFC, a concentric bipolar stimulating electrode (FHC; Bowdoinham, ME, USA) was placed in layer II of the PFC and a capillary glass recording electrode (Harvard Apparatus) filled with 3 M NaCl was placed in layer IV. Stimulation intensity was adjusted to evoke a field excitatory postsynaptic potential (fEPSP) at approximately 50% of the maximal response. The long-term potentiation (LTP) was induced by a theta-burst stimulation (TBS) protocol consisting of four trains delivered at 10-s intervals, with each train consisting of bursts of five pulses at 100 Hz, repeated at 200 ms intervals, as previously described [28, 29]. Data acquisition and analysis were performed using pClamp software (version 10.3; Axon Instruments).

### Western blot assay

Brain tissues were dissected and homogenized in lysis buffer containing 1% Triton X-100, 0.1% SDS, 50 mM Tris-HCl (pH 7.5), 0.3 M sucrose, 5 mM EDTA, 2 mM sodium pyrophosphate, 1 mM sodium orthovanadate, and 1 mM phenylmethylsulfonyl fluoride, supplemented with a complete protease inhibitor cocktail. The homogenates were sonicated and subsequently centrifuged at 12,000 rpm for 30 min at 4 °C to obtain the supernatant. Protein concentrations were determined using the Bradford assay. Equal amounts of protein were then resolved by SDS-PAGE, transferred onto Immobilon-P membranes (Millipore), and blocked with 5% non-fat dry milk for 1 h at room temperature. Western blot analysis was performed using GAPDH antibody (1:10000; GeneTex), GABA_A_R alpha 5 antibody (1:2000; Alomone), GABA_B_R B1 antibody (1:2000; Cell Signaling), GABA_B_R B2 antibody (1:2000; Abcam), phospho-eIF4E (Ser209) antibody (1:2000; Cell Signaling), eIF4E antibody (1:5000; Cell Signaling). Membranes were incubated with primary antibodies overnight at 4 °C, followed by incubation with HRP-conjugated secondary antibodies for 1 h at room temperature. Immunoreactive bands were visualized using ECL Plus detection reagent (PerkinElmer, Boston, MA, USA). Films were exposed for varying durations to achieve optimal signal intensity without saturation. Band intensities were quantified by densitometric analysis using ImageJ software (NIH, Bethesda, MD, USA). Protein expression levels were first normalized to internal loading controls and then expressed as fold changes relative to the control group.

### Drug treatment and local infusion

To validate resistance to antidepressant treatment, the mice received an intraperitoneal injection of fluoxetine (10 mg/kg), imipramine (10 mg/kg), and venlafaxine (10 mg/kg). Fluoxetine (10 mg/kg) was administered for 21 d prior to all depressive-like behavior tests [30]. Imipramine (10 mg/kg) was administered for 14 d prior to all depressive-like behavior tests [31]. Venlafaxine (10 mg/kg) was administered for 21 d prior to all depressive-like behavior tests [32]. To examine the effects of the target molecule on cognitive and depression-like behaviors, the eIF4E–eIF4G inhibitor 4EGI-1 was dissolved in vehicle solution of 60% dimethyl sulfoxide to final concentration of 5 μg/μL (1.25 μg in 0.25 μL per side). The 40-week-old mice were anesthetized with Zoletil & Rompun mix (0.1 mL/10 g). Mice were secured in a stereotaxic apparatus and infused 4EGI-1 (1.25 μg/side) into the PFC (anteroposterior, +1.7 mm, mediolateral, ±0.3 mm, dorsoventral, −2.3 mm). The mice were returned to their home cages for a week. Muscimol, a GABA_A_R agonist, (0.1 mg/kg i.p.) and baclofen, a GABA_B_R agonist, (1 mg/kg i.p.) were injected 60 min before the behavioral test.

### Ingenuity pathway analysis

Ingenuity Pathway Analysis (IPA) was performed using microarray datasets obtained from the Gene Expression Omnibus (GEO), including peripheral blood samples from patients with treatment-resistant depression (TRD) (GSE45468 and GSE199536), as well as PFC tissue from mouse models of depression, specifically the chronic unpredictable stress model (GSE145970) and the chronic social defeat stress model (GSE146845). Differentially expressed genes from each dataset were analyzed using the IPA software (QIAGEN, Hilden, Germany). Pathways belonging to the Canonical Pathway Analysis and Upstream Regulator Comparison Analysis section were considered for the analysis. Pathways with a -log10 (p-value) ≥ 1.3 (corresponding to p < 0.05) were considered statistically significant. The heat maps were generated by Morpheus.

### Statistical analysis

All values are expressed as the mean ± standard error of mean (SEM). Differences between the groups assessed using the *t*-test, Kolmogorov–Smirnov test, one-way ANOVA, and two-way ANOVA. Bonferroni *post hoc* comparison was used to analyze the differences in the results of the behavioral tests, electrophysiological responses, and protein levels between the control and traumatic stress groups. Probability values (*p*) < 0.05 were considered to represent significant differences.

## Results

### Antidepressant resistant symptoms were observed after traumatic stress exposure

**Fig. 1:**
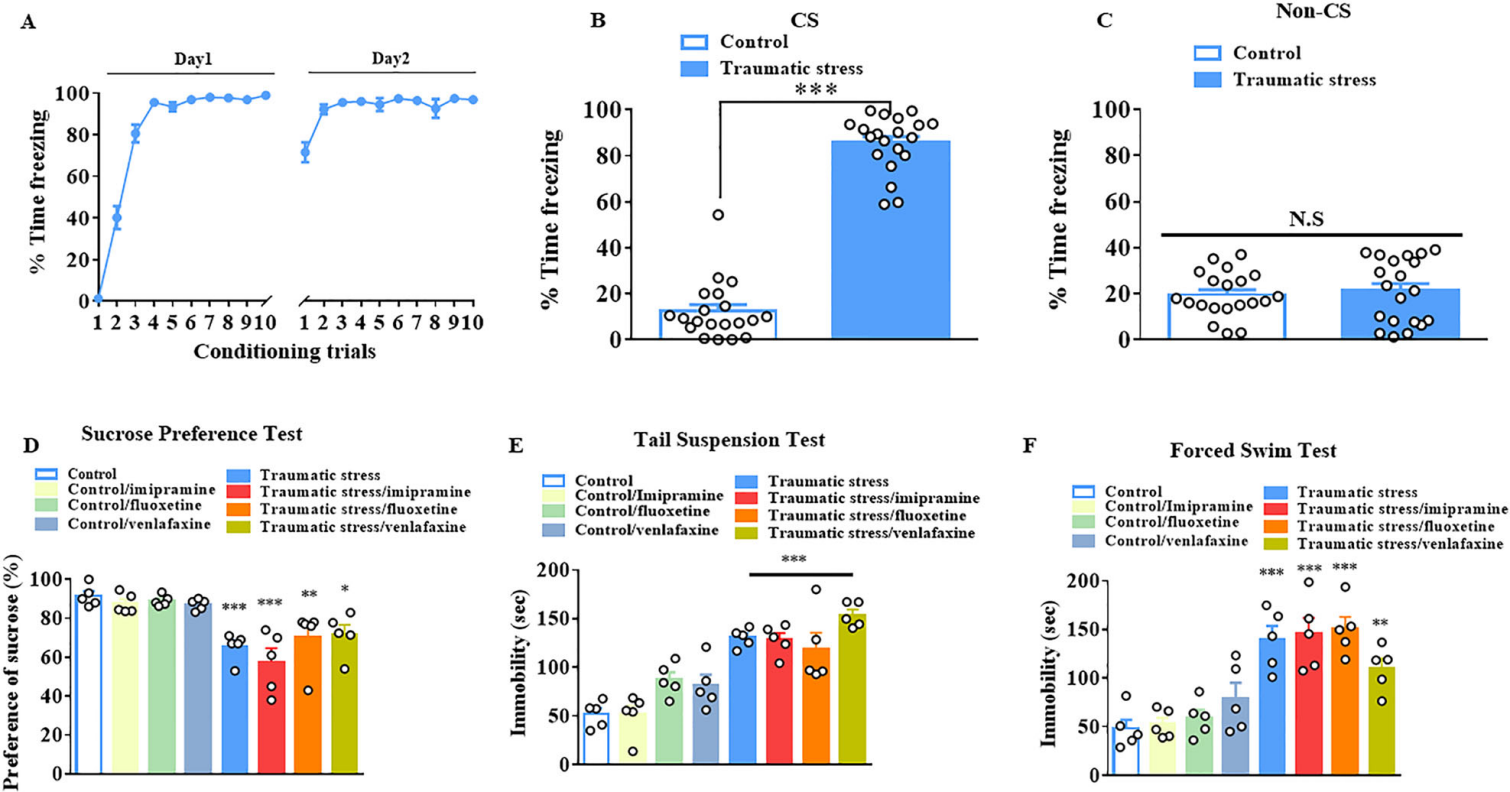
Typical antidepressant treatment for traumatic stress exposure-induced depressive-like behavior. (**A**) The freezing response during each fear-conditioning trial. **B** The freezing response with CS. **C** The freezing response without CS. **D** The sucrose preference following fluoxetine, imipramine, and venlafaxine treatment in control and traumatic stress-exposed mice (n = 5 in each group). **E** Immobility duration in the tail suspension test following fluoxetine, imipramine, and venlafaxine treatments in control and traumatic stress-exposed mice (n = 5 in each group). **F** Immobility duration in the forced swim test following fluoxetine, imipramine, and venlafaxine treatments in control and traumatic stress-exposed mice (each group n = 5). Data are represented as the mean ± SEM in each experiment. * *p* < 0.05, ** *p* < 0.01, *** *p* < 0.001 each group were compared by one-way ANOVA with Bonferroni *post-hoc* test. CS, conditioned stimulus; SEM, standard error of the mean; ANOVA, analysis of variance.

In the present study, we modified a previously established rat model that exhibits antidepressant resistant symptoms [28, 29]. The mouse model was induced by two days of foot shock to mimic traumatic stress. The fear test was conducted with or without a CS approximately 24 h after foot shock. The percentage of freezing time increased following conditioning trials (Fig. 1A). The freezing response showed a significant increase after 2 d of traumatic stress with CS presentation in traumatic stress exposure mice (*t*_(38)_ = 18.62; *p* < 0.001, n = 20 in each group; Fig. 1B). There was no significant difference in the percentage of freezing response between the groups without CS presentation (*t*_(38)_ = 0.437; *p* = 0.4784, n = 20 in each group; Fig. 1C). Next, control and traumatic stress exposed mice were evenly distributed into four groups: no treatment, fluoxetine treatment, imipramine treatment, and venlafaxine treatment. Behavioral tests were performed on day 15 of treatment for the imipramine-treated group, and on day 22 of treatment for both the fluoxetine- and venlafaxine-treated groups. The sucrose preference results showed no significant difference in control mice after imipramine, fluoxetine, and venlafaxine treatment (*F*_(7,32)_ = 9.091; control/imipramine: *p* > 0.99 vs control; control/fluoxetine: *p* > 0.99 vs control; control/venlafaxine: *p* > 0.99 vs control, *n* = 5 in each group; Fig. 1D). Traumatic stress exposed mice exhibited a significant reduction in sucrose preference, irrespective of whether they were untreated or treated with fluoxetine, imipramine, or venlafaxine (traumatic stress: *p* < 0.001 vs control; traumatic stress/imipramine: *p* < 0.001 vs control; traumatic stress/fluoxetine: *p* < 0.01 vs control; traumatic stress/venlafaxine: *p* < 0.05 vs control, *n* = 5 in each group; Fig. 1D). Moreover, there was no significant improvement after imipramine, fluoxetine, and venlafaxine treatment in traumatic stress-exposed mice (traumatic stress: *p* > 0.99 vs traumatic stress; traumatic stress/imipramine: *p* > 0.99 vs traumatic stress; traumatic stress/fluoxetine: *p* > 0.99 vs traumatic stress; traumatic stress/venlafaxine: *p* > 0.99 vs traumatic stress; Fig. 1D). The TST results showed no significant difference in the control mice after imipramine, fluoxetine, and venlafaxine treatments (*F*_(7,32)_ = 17.09; control/imipramine: *p* > 0.99 vs control; control/fluoxetine: *p* > 0.0739 vs control; control/venlafaxine: *p* > 0.2126 vs control, *n* = 5 in each group; Fig. 1E). Traumatic stress exposed mice exhibited a significant increase in immobility, irrespective of whether they were untreated or treated with fluoxetine, imipramine, or venlafaxine (traumatic stress: *p* < 0.001 vs control; traumatic stress/imipramine: *p* < 0.001 vs control; traumatic stress/fluoxetine: *p* < 0.001 vs control; traumatic stress/venlafaxine: *p* < 0.001 vs control, *n* = 5 in each group; Fig. 1E). Furthermore, there was no significant change after imipramine, fluoxetine, and venlafaxine treatment in traumatic stress-exposed mice (traumatic stress: *p* > 0.99 vs traumatic stress; traumatic stress/imipramine: *p* > 0.99 vs traumatic stress; traumatic stress/fluoxetine: *p* > 0.99 vs traumatic stress; traumatic stress/venlafaxine: *p* > 0.99 vs traumatic stress; Fig. 1E). The FST results showed no significant difference in the control mice after imipramine, fluoxetine, and venlafaxine treatment (*F*_(7,32)_ = 0.784; control/imipramine: *p* > 0.99 vs control; control/fluoxetine: *p* > 0.99 vs control; control/venlafaxine: *p* > 0.99 vs control, *n* = 5 in each group; Fig. 1F). Traumatic stress exposed mice exhibited a significant increase in immobility, irrespective of whether they were untreated or treated with fluoxetine, imipramine, or venlafaxine (traumatic stress: *p* < 0.001 vs control; traumatic stress/imipramine: *p* < 0.001 vs control; traumatic stress/fluoxetine: *p* < 0.001 vs control; traumatic stress/venlafaxine: *p* < 0.01 vs control, *n* = 5 in each group; Fig. 1F). Furthermore, there was no significant change after imipramine, fluoxetine, and venlafaxine treatments in traumatic stress-exposed mice (traumatic stress: *p* > 0.99 vs traumatic stress; traumatic stress/imipramine: *p* > 0.99 vs traumatic stress; traumatic stress/fluoxetine: *p* > 0.99 vs traumatic stress; traumatic stress/venlafaxine: *p* > 0.99 vs traumatic stress; Fig. 1F).

### Depression-like behaviors were observed after traumatic stress in both young and middle-aged mice

**Fig. 2:**
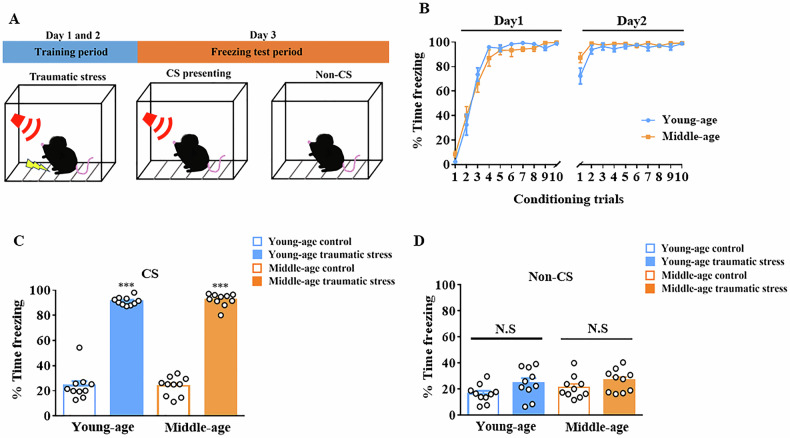
Freezing response of traumatic stress in young and middle-aged mice. (**A**) Time frame of traumatic stress. **B** Freezing response during each fear-conditioning trial (each group n = 10). **C** Freezing response with CS (each group n = 10). **D** Freezing response without CS (each group n = 10). Data are represented as the mean ± SEM, *** *p* < 0.001 each group were compared by two-way ANOVA. CS, conditioned stimulus; SEM, standard error of the mean; ANOVA, analysis of variance.

**Fig. 3:**
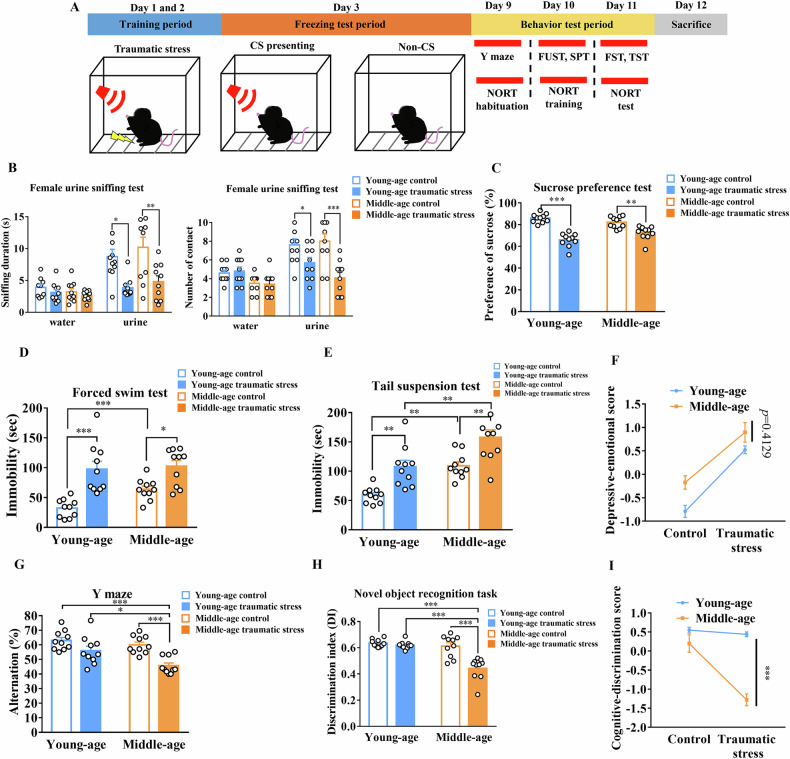
Depressive-like behavior and cognitive behavior after traumatic stress in young and middle-aged mice. (**A**) Time frame of experiment. **B** The duration of sniffing and the number of contact encounters with female urine after traumatic stress exposure (each group n = 10). **C** Percentage of sucrose preference after traumatic stress exposure (each group n = 10). **D** Immobility time of FST after traumatic stress exposure (each group n = 10). **E** Immobility time of TST after traumatic stress (each group n = 10). **F** Interaction analysis of depressive-emotional score (each group n = 10). **G** Percentage of spontaneous alteration Y-maze after traumatic stress exposure (each group n = 10). **H** DI of NORT after traumatic stress exposure (each group n = 10). **I** Interaction analysis of cognitive-discrimination score (each group n = 10). Data are represented as the mean ± SEM, * *p* < 0.05, ** *p* < 0.01, *** *p* < 0.001 each group were compared by two-way ANOVA. SEM, standard error of the mean; ANOVA, analysis of variance; FST, forced swim test; TST, tail suspension test; DI, discrimination index; NORT, novel object recognition task.

The freezing time percentage increased following conditioning trials in both young and middle-aged mice (Fig. 2A and B). The freezing response showed a significant increase after 2 d of traumatic stress exposure with CS presentation in both young and middle-aged mice (*F*_(1,36)_ = 232.1; young-age: *p* < 0.001 vs. young-age control; middle-age: *p* < 0.001 vs. middle-age control, n = 10 in each group; Fig. 2C). Moreover, there was no significant difference in each group without CS presentation in either young or middle-aged mice (*F*_(1,36)_ = 2.384; young-age: *p* = 0.3867 vs. young-age control; middle-age: *p* = 0.1584 vs. middle-age control, n = 10 in each group; Fig. 2D). Next, we examined depressive-like behavior after traumatic stress exposure in young and middle-aged mice (Fig. 3A). In the FUST, the sniffing duration and contact time were significantly decreased after traumatic stress exposure in both young- and middle-aged mice (sniffing duration: *F*_(3,72)_ = 7.703; young-age: *p* < 0.05 vs. young-age control; middle-age: *p* < 0.01 vs. middle-age control, n = 10 in each group; number of contact: *F*_(3,72)_ = 7.709; young-age: *p* < 0.05 vs. young-age control; middle age: *p* < 0.001 vs. middle-age control, n = 10 in each group; Fig. 3B). Sucrose preference significantly decreased after traumatic stress exposure in both the young- and middle-aged group (*F*_(1,36)_ = 70.35; young-age: *p* < 0.001 vs. young-age control; middle-age: *p* < 0.01 vs. middle-age control, n = 10 in each group; Fig. 3C). The FST results showed that immobility time significantly increased after traumatic stress exposure in both the young and middle-aged group (*F*_(1,36)_ = 32.97; young-age: *p* < 0.001 vs. young-age control; middle-age: *p* < 0.05 vs. middle-age control, n = 10 in each group; Fig. 3D). Moreover, in the middle-aged control group, immobility time was significantly increased (*p* < 0.001 vs. young-age control; Fig. 3D). Similarly, the TST results indicated that the immobility time significantly increased after traumatic stress exposure in both the young and middle-aged group (*F*_(1,36)_ = 27.02; young-age: *p* < 0.01 vs. young-age control; middle-age: *p* < 0.01 vs. middle-age control, n = 10 in each group; Fig. 3E). Moreover, in the control group, immobility time significantly increased in the middle-aged group (*p* < 0.01 vs. young-age control; Fig. 3E). However, no significant interaction between aging and traumatic stress effect was observed in depressive-emotional score (*F*_(1,36)_ = 0.6861; *p* = 0.4129, n = 10 in each group; Fig. 3F).

### Traumatic stress-induced cognitive impairment was only observed in middle-aged mice

The spontaneous alteration Y-maze test and NORT were performed to investigate cognitive function in TRD after aging. The spontaneous alteration of Y-maze results indicated that the percentage of spontaneous alterations significantly decreased after traumatic stress exposure in the middle-aged group (*F*_(1,36)_ = 21.58; *p* < 0.001 vs. young-age control; *p* < 0.001 vs. middle-age control, n = 10 in each group; Fig. 3G). Moreover, the percentage of spontaneous alterations significantly decreased in the middle-aged group compared with young-aged mice after traumatic stress (*p* < 0.05 vs. young-aged traumatic stress; Fig. 3G). The NORT results also demonstrated that the discrimination index (DI) significantly decreased after traumatic stress exposure in the middle-aged group (*F*_(1,36)_ = 21.72; *p* < 0.001 vs. young-age control; *p* < 0.001 vs. middle-age control, n = 10 in each group; Fig. 3H), while the DI significantly decreased in the middle-aged group compared to young-aged after traumatic stress (*p* < 0.001 vs. young-aged traumatic stress; Fig. 3H). Interestingly, a significant interaction between aging and the effect of traumatic stress was found in the cognitive-discrimination score (*F*_(1,36)_ = 21.88; *p* = 0.001, n = 10 in each group; Fig. 3I). The evidence suggests a significant interaction between age and cognitive function following traumatic stress.

### Synaptic plasticity was aberrant after traumatic stress in both young and middle-aged mice

**Fig. 4:**
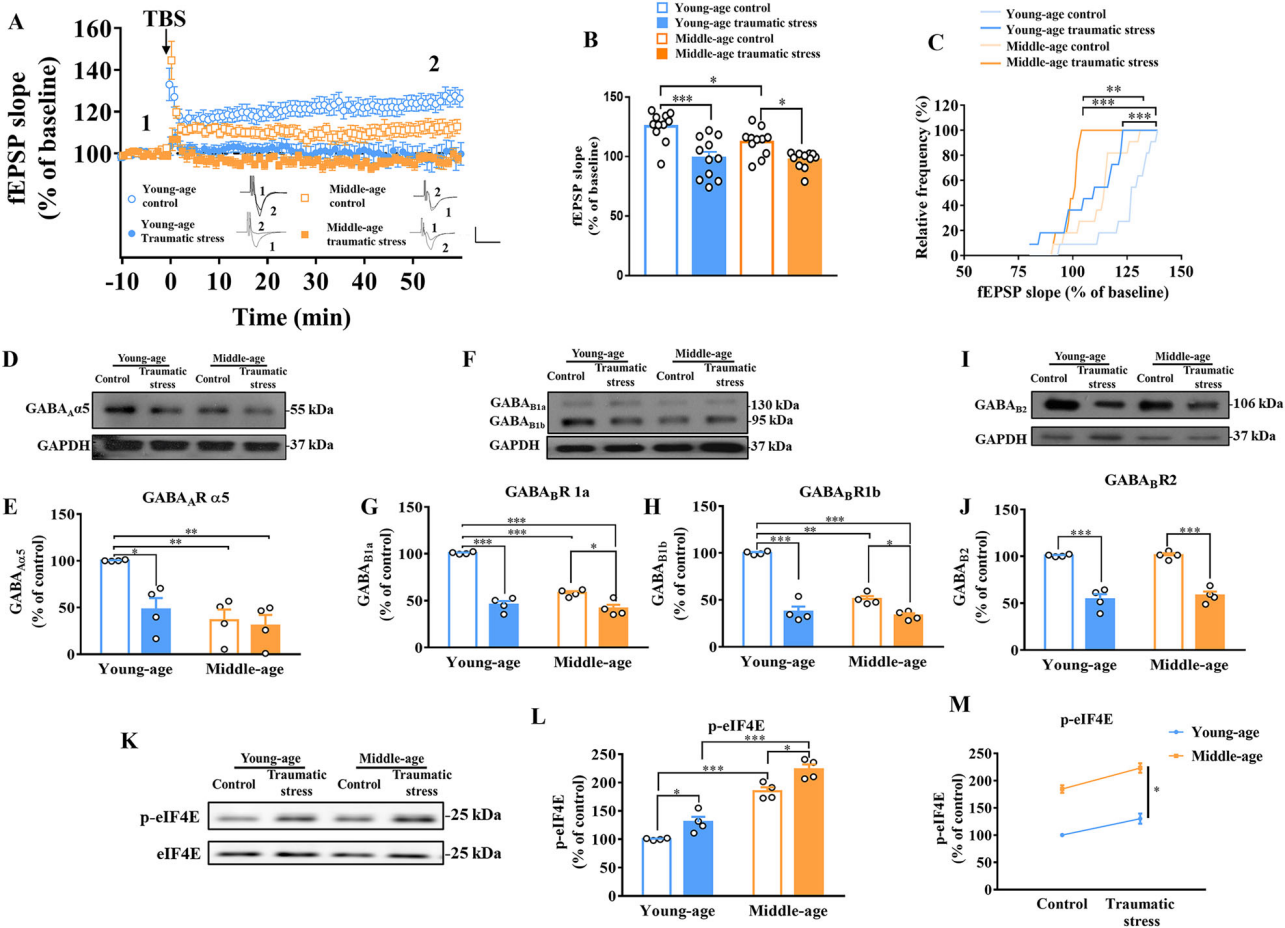
Synaptic plasticity, expression of GABAR and phosphorylation of eIF4E after traumatic stress in young and middle-aged mice. (**A**) The fEPSP slope during LTP induction by TBS (each group n = 6 slices from 4 mice; scale: 0.5 mV/10 ms). **B** The average fEPSP slope of last 10 min during LTP induction in each group (each group n = 11 slices from 6 mice). **C** The cumulative probability of each group (each group n = 11 slices from 6 mice). **D** Representative GABA_A_R α5 subunit expression by Western blot in PFC. **E** Representative Western blot and summary bar graph of GABA_A_R α5 subunit (each group n = 4). **F** Representative GABA_B_R1 subunits expression by Western blot in PFC. **G** Representative Western blot and summary bar graph of the GABA_B_R1a (each group n = 4). **H** Representative Western blot and summary bar graph of the GABA_B_R1b (each group n = 4). **I** Representative GABA_B_R2 subunit expression by Western blot in PFC. **J** Representative Western blot and summary bar graph of GABA_B_R2 (each group n = 4). **K** Representative eIF4E phosphorylation and expression by Western blot in PFC. **L** Phosphorylation levels of eIF4E (each group n = 4). **M** Interaction analysis of phosphorylation levels of eIF4E. Data are represented as the mean ± SEM, * *p* < 0.05, ** *p* < 0.01, *** *p* < 0.001 each group was compared by one-way ANOVA, Kolmogorov–Smirnov test and two-way ANOVA. fEPSP, field excitatory postsynaptic potential; LTP, long-term potentiation; TBS, theta-burst stimulation; GABAR, γ-aminobutyric acid receptor; eIF4E; eukaryotic translation initiation factor; PFC, prefrontal cortex; SEM, standard error of the mean; ANOVA, analysis of variance.

Next, TBS-induced LTP was applied to the PFC to measure synaptic plasticity. Impaired LTP was observed after traumatic stress exposure in both the young and middle-aged groups (*F*_(1,40)_ = 29.51; young-age: *p* < 0.001 vs. young-age control; middle-age: *p* < 0.05 vs. middle-age control, n = 11 in each group; Fig. 4A and B). In the control group, LTP was impaired in the middle-aged group (*p* < 0.05 versus young-age control; Fig. 4A and B). However, no significant differences were observed between the young and middle-aged groups after traumatic stress exposure. Similarly, a significant leftward shift in cumulative probability was observed after traumatic stress exposure in both the young and middle-aged groups (young-age: *p* < 0.001 vs. young-age control; middle-age: *p* < 0.01 vs. middle-age control, n = 11 in each group; Fig. 4C), indicating that aberrant synaptic plasticity was correlated with traumatic stress, whereas aging did not worsen impairment after traumatic stress exposure.

### Alterations in GABA_A_R and GABA_B_R expression and eIF4E phosphorylation were observed after traumatic stress in both the young and middle-aged group

To investigate whether age and depression impact the GABAergic system, the expression of the GABAR subunits in the PFC was examined. The expression levels of the GABA_A_R α5 subunit significantly decreased after traumatic stress in the young- and middle-aged group (*F*_(1,12)_ = 8.222; young-age: *p* < 0.05 vs. young-age control; middle-age: *p* < 0.01 vs. young-age control, n = 4 in each group; Fig. 4D and E). Moreover, decreased GABA_A_R α5 subunit expression was observed in the middle-aged control group (*p* < 0.01 vs. young-age control, n = 4 in each group; Fig. 4D and E). However, there was no significant change in middle-aged mice with or without traumatic stress. GABA_B_R1a subunit expression significantly decreased after traumatic stress exposure in young and middle-aged mice (*F*_(1,12)_ = 147.3; young-age: *p* < 0.001 vs. young-age control; middle-age: *p* < 0.05 vs. middle-age control, n = 4 in each group; Fig. 4F and G). Decreased GABA_B_R1a subunit expression was also observed in the middle-aged control group (*p* < 0.001 vs. young-age control, n = 4 in each group; Fig. 4F and G). Similarly, GABA_B_R1b subunit expression was significantly decreased after traumatic stress exposure in young- and middle-aged mice (*F*_(1,12)_ = 137.7; young-age: *p* < 0.001 vs. young-age control; middle-age: *p* < 0.05 vs. middle-age control, n = 4 in each group; Fig. 4F and H). Decreased GABA_B_R1b subunit expression was also observed in the middle-aged control group (*p* < 0.01 vs. young-age control, n = 4 in each group; Fig. 4F and H). GABA_B_R2 subunits expression was significantly decreased after traumatic stress in the young- and middle-aged groups (*F*_(1,12)_ = 148.2; young-age: *p* < 0.001 vs. young-age control; middle-age: *p* < 0.001 vs. middle-age control, n = 4 in each group; Fig. 4I and J). Next, the phosphorylated form of eIF4E in the PFC was examined to evaluate the effects of age and depression on eIF4E phosphorylation. The phosphorylation of eIF4E was significantly increased in the middle-aged control group (*F*_(1,12)_ = 22.27; *p* < 0.001 vs. young-age control, n = 4 in each group; Fig. 4K, L). eIF4E phosphorylation further increased after traumatic stress exposure in the middle-aged group (*p* < 0.05 vs. middle-age control; Fig. 4K, L). In addition, increased eIF4E phosphorylation showed a significant effect of age after traumatic stress (*F*_(1,12)_ = 148.8; *p* < 0.05, n = 4 in each group; Fig. 3M).

### Traumatic stress-induced depressive-like behavior and cognitive impairment were improved by inhibition of eIF4E phosphorylation

**Fig. 5:**
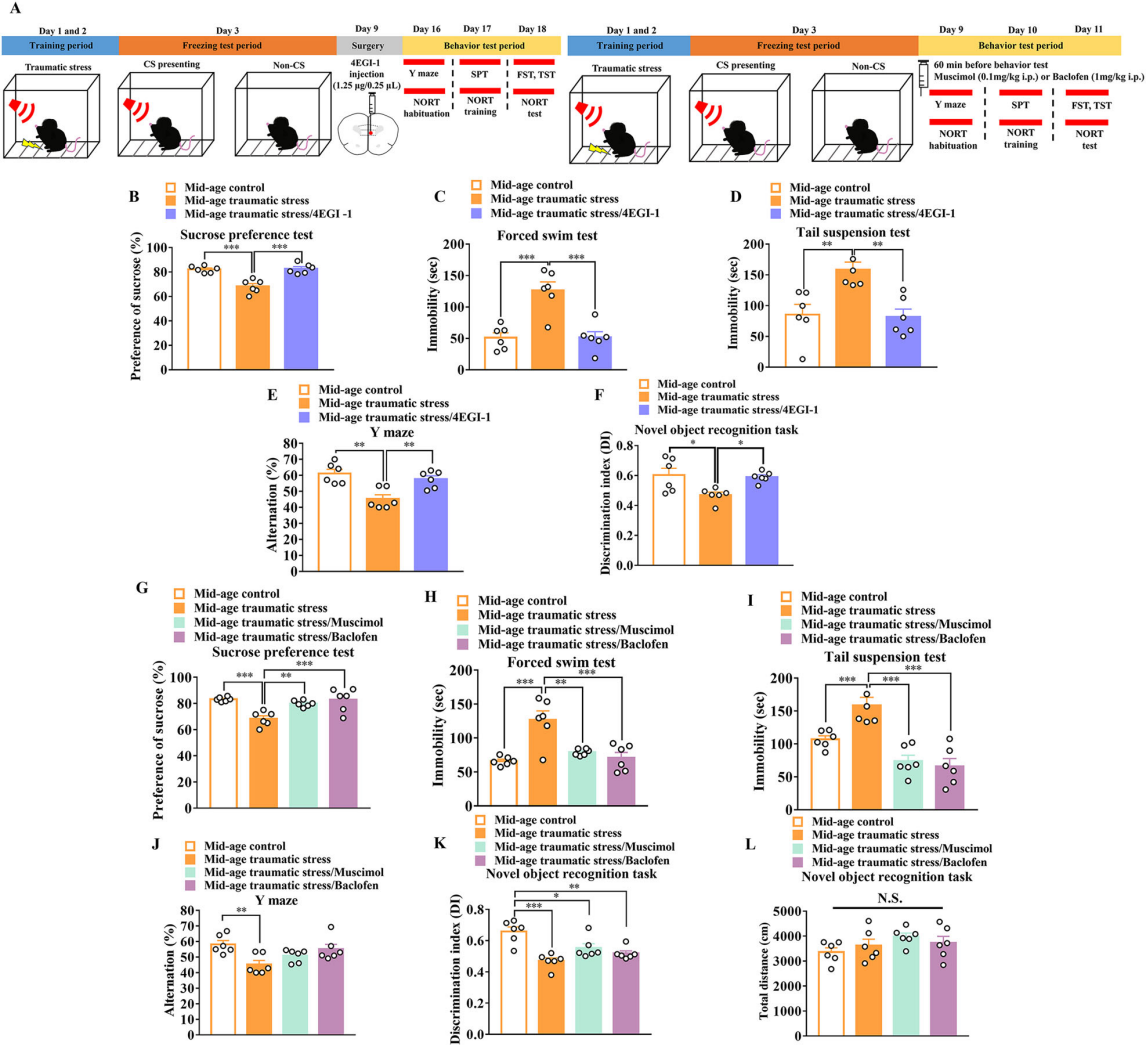
Depressive-like behavior and cognitive behavior of traumatic stress after 4EGI-1 infusion or muscimol or baclofen treatment in middle-aged mice. (**A**) Time frame of experiment. **B** Percentage of sucrose preference after 4EGI-1 infusion in middle-aged traumatic stress mice (each group n = 6). **C** Immobility time of FST after 4EGI-1 infusion in middle-aged traumatic stress mice (each group n = 6). **D** Immobility time of TST after 4EGI-1 infusion in middle-aged traumatic stress mice (each group n = 6). **E** Percentage of spontaneous alteration Y-maze after 4EGI-1 infusion in middle-aged traumatic stress mice (each group n = 6). **F** DI of NORT after 4EGI-1 infusion in middle-aged traumatic stress mice (each group n = 6). **G** Percentage of sucrose preference after muscimol or baclofen treatment in middle-aged traumatic stress mice (each group n = 6). **H** Immobility time of FST after muscimol or baclofen treatment in middle-aged traumatic stress mice (each group n = 6). **I** Immobility time of TST after muscimol or baclofen treatment in middle-aged traumatic stress mice (each group n = 6). **J** Percentage of spontaneous alteration Y-maze after muscimol or baclofen treatment in middle-aged traumatic stress mice (each group n = 6). **K** DI of NORT after muscimol or baclofen treatment in middle-aged traumatic stress mice (each group n = 6). **L** Total distance of NORT after muscimol or baclofen treatment in middle-aged traumatic stress mice (each group n = 6). Data are represented as the mean ± SEM. * *p* < 0.05, ** *p* < 0.01, *** *p* < 0.001 each group was compared by one-way ANOVA. SEM, standard error of the mean; ANOVA, analysis of variance; FST, forced swim test; TST, tail suspension test; DI, discrimination index; NORT, novel object recognition task.

To determine whether elevated eIF4E phosphorylation leads to cognitive dysfunction in midlife depression, 4EGI-1, a competitive eIF4E/eIF4G interaction inhibitor, in the PFC of middle-aged mice was used (Fig. 5A). The SPT results indicated that the decreased sucrose preference after traumatic stress exposure was improved by 4EGI-1 administration (*F*_(2,15)_ = 22.27; *p* < 0.001 vs. middle-age traumatic stress, n = 6 in each group; Fig. 5B). Increased immobility time in the FST after traumatic stress exposure was improved by 4EGI-1 administration (*F*_(2,15)_ = 17.67; *p* < 0.001 vs. middle-age traumatic stress, n = 6 in each group; Fig. 5C). Increased immobility time in the TST after traumatic stress exposure improved following 4EGI-1 administration (*F*_(2,15)_ = 9.75; *p* < 0.01 versus middle-age traumatic stress, n = 6 in each group; Fig. 5D). Furthermore, the spontaneous alteration Y-maze results indicated that the decreased percentage of alteration after traumatic stress exposure was improved by 4EGI-1 administration (*F*_(2,15)_ = 11.42; *p* < 0.01 vs. middle-age traumatic stress, n = 6 in each group; Fig. 5E). The NORT indicated that the decreased DI after traumatic stress exposure was improved by 4EGI-1 administration (*F*_(2,15)_ = 6.101; *p* < 0.05 vs. middle-age traumatic stress, n = 6 in each group; Fig. 5F), indicating that eIF4E phosphorylation plays a role in depressive symptoms and cognitive function.

### Traumatic stress-induced depression-like behavior was improved by activation of GABAR

To determine whether GABA_A_R and GABA_B_R are involved in cognitive dysfunction in midlife depression, muscimol, a GABA_A_R agonist, and baclofen, a GABA_B_R agonist, were administered to middle-aged mice (Fig. 5A). The SPT results indicated that decreased sucrose preference after traumatic stress exposure was improved by treatment with muscimol and baclofen (*F*_(3,20)_ = 10.05; muscimol*: p* < 0.01 vs. middle-age traumatic stress; baclofen*: p* < 0.001 vs. middle-age traumatic stress, n = 6 in each group; Fig. 5G). The increased immobility time in the FST after traumatic stress was improved by treatment with muscimol and baclofen (*F*_(3,20)_ = 12.37; muscimol*: p* < 0.01 vs. middle-age traumatic stress; baclofen*: p* < 0.001 vs. middle-age traumatic stress, n = 6 in each group; Fig. 5H). The increased immobility time in the TST after traumatic stress was improved by treatment with muscimol and baclofen (*F*_(3,20)_ = 17.57; muscimol*: p* < 0.001 vs. middle-age traumatic stress; baclofen*: p* < 0.001 vs. middle-age traumatic stress; n = 6 in each group; Fig. 5I). However, the spontaneous alteration of Y-maze results indicated that the decreased alteration percentage after traumatic stress was not improved by muscimol or baclofen treatment (*F*_(3,20)_ = 5.096; muscimol*: p* = 0.746 vs. middle-age traumatic stress; baclofen*: p* = 0.065 vs. middle-age traumatic stress; n = 6 in each group; Fig. 5J). The NORT indicated that the decreased DI after traumatic stress exposure was not improved by muscimol or baclofen treatment (*F*_(3,20)_ = 11.36; muscimol*: p* = 0.817 vs. middle-age traumatic stress; baclofen*: p* = 0.916 vs. middle-age traumatic stress; n = 6 in each group; Fig. 5K), indicating that GABA_A_R and GABA_B_R levels were only implicated in depressive symptoms. The total distance during the NORT showed that there was no significant difference between the groups (*F*_(3,20)_ = 1.42; traumatic stress: *p* = 0.831 vs. middle-age control; muscimol*: p* = 0.210 vs. middle-age control; baclofen*: p* = 0.628 vs. middle-age control; n = 6 in each group; Fig. 5L).

### Canonical and comparison analysis highlights the involvement of eIF4E-related pathway in depression across human and animal studies

**Fig. 6:**
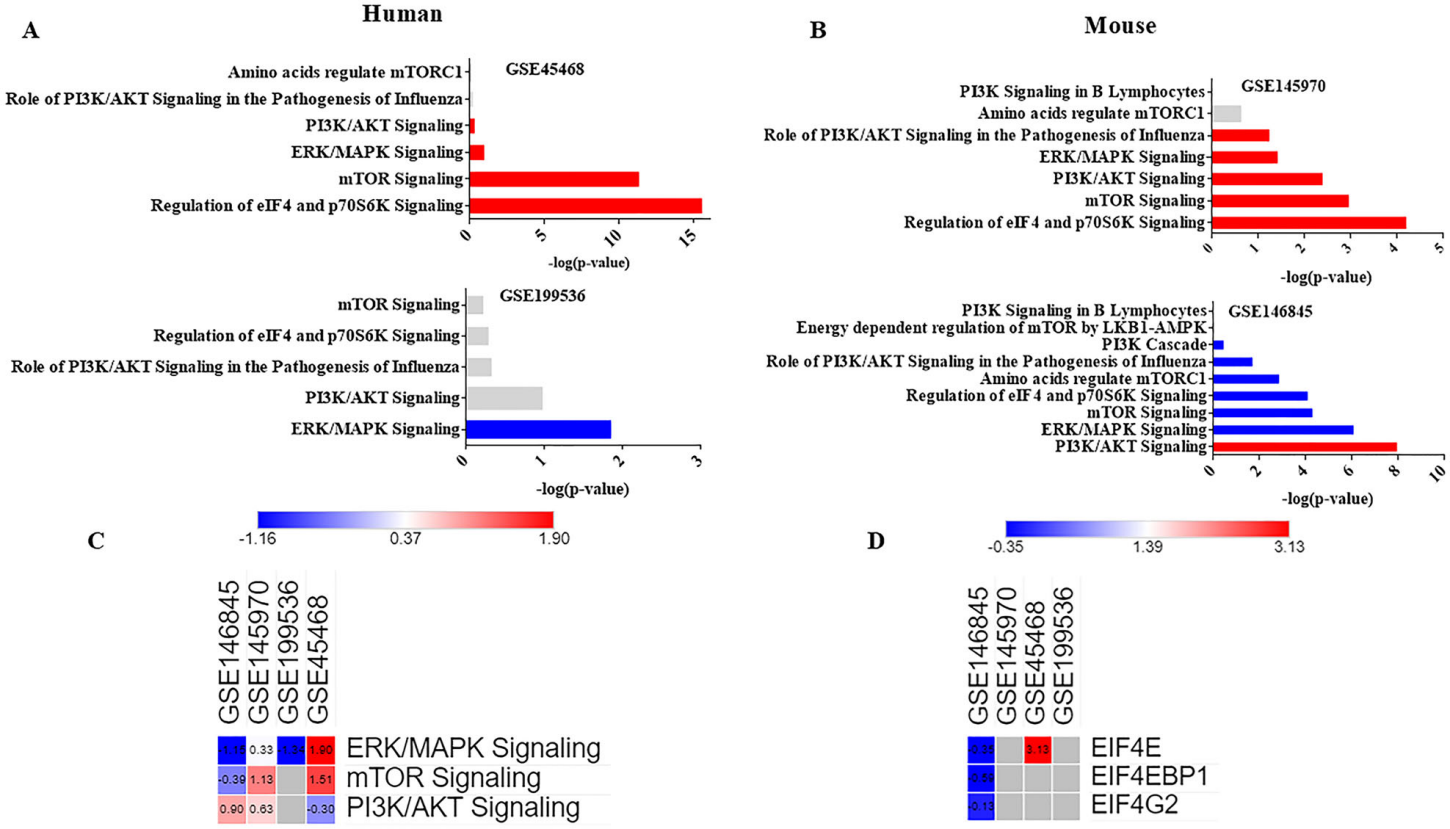
Comparative analysis of depressive-mice and human studies by IPA. (**A**) Canonical IPA analysis of differentially expressed genes in human studies. **B** Canonical IPA analysis of differentially expressed genes in mice studies. **C** Canonical pathway comparison analysis between mice and human studies. **D** Upstream regulator comparison analysis between mice and human studies. Positive z scores indicate an increase whereas negative z scores indicate a reduction in each group. IPA, ingenuity pathway analysis.

Ingenuity pathway analysis (IPA) was used to explore the association between eIF4E and depression in human and animal studies. We performed microarray analysis of peripheral blood samples from patients with TRD and a depression mouse model PFC sample from the GEO datasets. Canonical pathway analysis showed that the regulation of eIF4E-related signaling, including PI3K/AKT, ERK/MAPK, mTOR, and eIF4 and p70S6K signaling, was associated with TRD, as identified through the peripheral blood samples of patients with TRD (GSE45468 and GSE199536) microarray sample (Fig. 6A). Canonical pathway analysis also indicated that the regulation of eIF4E-related signaling, including PI3K/AKT, ERK/MAPK, mTOR, and eIF4 and p70S6K signaling, was associated with depression in animals, based on PFC samples from mice subjected to chronic unpredictable stress (GSE145970) and chronic social defeat stress-induced mice (GSE146845) (Fig. 6B). Canonical pathway comparison indicated that ERK/MAPK, mTOR, and PI3K/AKT signaling were predicted to be involved in both patients with TRD and depressed animals (Fig. 6C). Upstream comparison predicted the upstream regulator, the eIF4F complex, which comprises eIF4E. Downregulation of eIF4E in animals (GSE146845) and upregulation of eIF4E in patients with TRD (GSE45468) are demonstrated (Fig. 6D). Although the regulatory outcomes were not consistent in each dataset, the results indicate that eIF4E-related pathways play a crucial role in TRD.

## Discussion

This study demonstrated a relationship between cognitive dysfunction and depression, particularly in mice exhibiting a poor antidepressant response induced by traumatic stress, with aging. Our findings provide further evidence that eIF4E may be a key factor associated with cognitive dysfunction and depression in the context of aging. An effective therapeutic strategy for treating cognitive dysfunction in TRD is urgently needed and eIF4E may be a potential target. Cognitive dysfunction is a core feature of depression [33]. Moreover, late-life depression, particularly in TRD, may be a risk factor for prodrome of dementia and AD [34–36]. Furthermore, depression beginning in midlife has been linked to an increased risk of developing vascular dementia [37]. However, antidepressant treatment often does not improve cognitive impairment in patients with depression [38]. To investigate this, we established a mouse model, modified from our previous work, with a specific focus on middle-age [28, 29]. Antidepressant resistant symptoms of depression-like behavior were also observed after two days of traumatic stress exposure. Moreover, cognitive impairment was observed in the middle-aged group after experiencing traumatic stress. The cohort study with 20 years of follow-up indicated that consistently high depressive symptom trajectories increase the risk of developing dementia, which may associated with white matter damage [6]. Higher depressive symptom trajectories in young to mid-adulthood have been linked to accelerated brain aging by midlife [5]. Furthermore, extended periods of higher depressive symptoms starting in young adulthood may lead to poorer cognitive function in midlife [39], suggesting the severity of depression during midlife may influence the extent of cognitive impairment later in life. However, our study found no differences in depressive-like behavior in the young or middle-age group after traumatic stress exposure. Another preclinical study showed that, compared with naïve 3-month-old mice, naïve 18-month-old mice exhibited only hedonic deficits, with no signs of behavioral despair [40]. The evidence above suggests that while age may not be related to the severity of depressive symptoms, midlife depression is recognized as a significant factor contributing to the later development of dementia, underscoring the importance of early detection and intervention in mitigating long-term cognitive decline.

In this study, increased eIF4E phosphorylation levels were correlated with aging in mice exhibiting a poor antidepressant response induced by traumatic stress. These behavioral test results provide further evidence that eIF4E phosphorylation is involved in depressive symptoms and cognitive impairment in a middle-aged mice exhibiting a poor antidepressant response induced by traumatic stress. To ensure that the observed behavioral outcomes were not merely immediate emotional reactions to traumatic stress, we conducted behavioral testing one week after stress exposure. The eIF4E–eIF4G interaction inhibitor, 4EGI-1, was administered to investigate the role of eIF4E phosphorylation. Our results demonstrate that 4EGI-1 alleviates depression-like behaviors and cognitive deficits. Mice lacking eIF4E phosphorylation exhibited cognitive behavior impairment, as observed in the NORT and the object location memory task [21]. A marked increase in eIF4E phosphorylation levels following the severity of neurofibrillary tangle pathology was observed in post-mortem brains of patients with AD [22]. Another animal study indicated that complete Freund’s adjuvant-induced inflammation and pain response regulated by eIF4E phosphorylation were only observed in aged mice [41]. As mentioned above, eIF4E phosphorylation is highly correlated with cognitive function, particularly in elderly populations.

The eIF4E upstream regulator, an mTOR signaling pathway, is involved in effective treatment options for TRD, such as low-dose ketamine [18]. The eIF4E mutation for the genetic inhibition of eIF4E phosphorylation exhibited anxiety- and depression-like behaviors and impaired serotonin-induced excitatory synaptic activity in the PFC, which is involved in inflammatory responses [42]. Depressive-like behavior induced by lipopolysaccharides involves synaptic protein loss and TrkB/BDNF signaling defects due to eIF4E-associated translational dysregulation caused by increased eIF4E phosphorylation [43]. A post-stroke depression animal study indicated that increased eIF4E activity after stroke induces depressive-like behavior and neuroinflammation, resulting from insufficient miR34b-3p [44]. These findings indicate that both increased and reduced eIF4E phosphorylation is associated with depressive symptoms, which may be linked to inflammatory responses. Furthermore, eIF4E may be considered as a potential target for cognitive dysfunction and depressive symptoms.

Previous studies have reported reduced GABA levels and decreased expression of GABA_A_R α1 and α2 subunit mRNAs in the serum of patients with depression [45]. Additionally, postmortem studies have revealed reduced expression of the GABA_B_R subunits GABBR1 and GABBR2 in the lateral cerebellum of individuals with depression [46]. Furthermore, a reduction in GABA_A_R and GABA_B_R-mediated inhibition has been observed in patients with TRD [47]. Animal research has further shown that GABAergic synapses in γ2^+/−^ mice can be enhanced by ketamine, an effective treatment for TRD, in the PFC [48]. Consistent with these findings, our results demonstrated that traumatic stress-induced depressive-like behavior was ameliorated by both GABA_A_R and GABA_B_R activation. Therefore, we investigated the role of GABA_A_R and GABA_B_R agonists in traumatic stress-induced depression and cognitive impairment in middle-aged mice. Our findings revealed that both agonists significantly improved stress-induced depressive-like behaviors. Previous preclinical studies have also reported that a GABA_A_R α5 subunit-positive allosteric modulator (PAM) exerts anxiolytic and antidepressant effects in mice subjected to chronic stress [49]. Moreover, somatostatin-positive GABA neuron deficit-induced anhedonia-like behavior and anxiety-like behavior was rescued by a GABA_A_R α5 subunit-PAM [50]. In the clinical context, GABA_A_R PAM have been used to treat postpartum depression [51], while preclinical studies have demonstrated that GABA_B_R agonists and PAMs exert antidepressant-like effects in learned helplessness models [52, 53]. Although stress has been associated with reduced GABA receptor expression, activation of the remaining functional receptors may still enhance inhibitory signaling and contribute to the restoration of neural circuit balance. Such modulation may underlie the observed behavioral improvements, highlighting the potential of GABA receptor agonists even in the presence of stress-induced GABAergic deficits.

Conversely, an age-dependent decline in GABA concentration has been reported in a magnetic resonance spectroscopy study [14]. However, cognitive impairment was ameliorated by GABA_A_R and GABA_B_R activation. Interestingly, our results indicated that expression of the GABA_A_R α5 subunit and GABA_B_R1 but not GABA_B_R2 significantly decreased in middle-age and with traumatic stress exposure. A reduction in the GABA_A_R α5 and GABA_B_R B1 subunits has been associated with spatial memory impairment in aged rats [16], and similar a reduction in α5 subunit have also been reported in aged rats with memory deficits [54]. A previous study demonstrated that the GABA_A_R α5 subunit-PAM reversed both stress-induced and age-related working memory deficits, whereas diazepam, a classical GABA_A_R agonist, failed to improve age-related working memory decline in mice subjected to chronic stress [49]. Consistent with these findings, our data showed that administration of either GABA_A_R or GABA_B_R agonists did not improve traumatic stress-induced cognitive impairment in middle-aged mice. Furthermore, treatment with baclofen (1 mg/kg) has been reported to alleviate depression-like behavior in young mice [55], although dose of 2.5 mg/kg were required to improve cognitive impairment in aged APP/PS1 transgenic mice [56]. These findings suggest that restoring cognitive function in the context of depression and aging may depend on targeting specific GABA receptor subunits or achieving sufficient pharmacological activation, rather than general GABAergic enhancement alone. However, the role of GABA receptors in cognitive function across aging and stress conditions requires further investigation.

Our results suggest that synaptic plasticity is impaired after traumatic stress in both young and middle-aged mice. Furthermore, impaired synaptic plasticity was observed in the middle-aged control mice. Disordered synaptic plasticity is influenced by exposure to stress and psychological trauma, resulting in cognitive impairment and emotional dysfunction [57]. Aging also plays a causal role in changes in synaptic plasticity and cellular alterations that directly affect the mechanisms underlying plasticity [58]. A preclinical study further indicated that impairment in synaptic plasticity occurs at mid-age (7–10-month-old) [59]. Phosphorylation of eIF4E is essential for synaptic plasticity [60]. A previous study has suggested that synaptic activity-induces eIF4E phosphorylation, which triggers the release of translational repressors from the cap complex and contributes to LTP maintenance [61]. In our previous study involving rats, we found that synaptic plasticity impairment observed one week after traumatic stress exposure could be ameliorated by non-invasive magnetic stimulation, a treatment mechanism associated with mTOR signaling upstream of eIF4E [30]. Prior research has shown that the mTOR signaling pathway plays a critical role in synaptic plasticity [62]. Furthermore, eIF4E phosphorylation is essential for both synaptic plasticity and cognitive function [60, 63]. These findings raise the possibility that modulation of eIF4E, even after the onset of stress-induced synaptic plasticity deficits, may still influence neural outcomes, suggesting that dysregulated eIF4E phosphorylation may contribute to synaptic plasticity impairment during aging in depression and provides a rationale for targeting translational control mechanisms in delayed therapeutic interventions.

Although the present study has certain limitations, it provides important mechanisms underlying age-related cognitive impairment in features of poor antidepressant response. We identified eIF4E as a potential molecular factor closely associated with both cognitive dysfunction and depressive symptoms in middle-age. To better understand the underlying mechanisms, we further refined and validated our previously established model of stress-induced features of poor antidepressant response in mice.

In our previous study, we applied mild, moderate, and severe levels of traumatic stress submit to rats and found that severe traumatic stress induced features of poor antidepressant response [28]. In the present study, we validated this experimental approach in mice using the same drug dosage and treatment duration as in previous animal studies [30–32]. We found that two days of foot-shock exposure as a traumatic stress also resulted in features of poor antidepressant response. While the drug treatment paradigm effectively captures core behavioral phenotypes, it may not fully replicate the chronic and progressive nature of TRD observed in clinical settings. According to the U.S. Food and Drug Administration and the American Psychiatric Association guidelines, antidepressant treatment in clinical practice typically requires 6 to 8 weeks to adequately evaluate therapeutic efficacy. Based on these guidelines, ongoing validation and refinement will help further align the animal model with the clinical features of TRD.

## Data Availability

The microarray datasets analyzed in this study are publicly available in the NCBI GEO database under accession numbers GSE45468, GSE199536, GSE145970, and GSE146845.
